# A descriptive analysis of perceptions of HIV risk and worry about acquiring HIV among FEM-PrEP participants who seroconverted in Bondo, Kenya, and Pretoria, South Africa

**DOI:** 10.7448/IAS.17.3.19152

**Published:** 2014-09-08

**Authors:** Amy L Corneli, Kevin McKenna, Jennifer Headley, Khatija Ahmed, Jacob Odhiambo, Joseph Skhosana, Meng Wang, Kawango Agot, for the FEM-PrEP Study Group

**Affiliations:** 1 Social and Behavioral Health Sciences, FHI 360, Durham, NC, USA; 2 Setshaba Research Centre, Soshanguve, South Africa; 3 Impact Research and Development Organization, Kisumu, Kenya; 4 Quantitative Sciences, Durham, NC FHI 360, USA

**Keywords:** risk perceptions, HIV worry, FEM-PrEP, seroconversion, women, Africa

## Abstract

**Introduction:**

Risk perception is a core construct in many behaviour change theories in public health. Individuals who believe they are at risk of acquiring an illness may be more likely to engage in behaviours to reduce that risk; those who do not feel at risk may be unlikely to engage in risk reduction behaviours. Among participants who seroconverted in two FEM-PrEP sites – Bondo, Kenya, and Pretoria, South Africa – we explored perceived HIV risk and worry about acquiring HIV prior to HIV infection.

**Methods:**

FEM-PrEP was a phase III clinical trial of once-daily, oral emtricitabine and tenofovir disoproxil fumarate for HIV prevention among women in sub-Saharan Africa. We asked all participants about their perceived HIV risk in the next four weeks, prior to HIV testing, during a quantitative face-to-face interview at enrolment and at quarterly follow-up visits. Among participants who seroconverted, we calculated the frequencies of their responses from the visit conducted closest to, but before, HIV acquisition. Also among women who seroconverted, we conducted qualitative, semi-structured interviews (SSIs) at weeks 1, 4 and 8 after participants’ HIV diagnosis visit to retrospectively explore feelings of HIV worry. Applied thematic analysis was used to analyse the SSI data.

**Results:**

Among participants who seroconverted in Bondo and Pretoria, 52% reported in the quantitative interview that they had no chance of acquiring HIV in the next four weeks. We identified four processes of risk rationalization from the SSI narratives. In “protective behaviour,” participants described at least one risk reduction behaviour they used to reduce their HIV risk; these actions made them feel not vulnerable to HIV, and therefore they did not worry about acquiring the virus. In “protective reasoning,” participants considered their HIV risk but rationalized, based on certain events or beliefs, that they were not vulnerable and therefore did not worry about getting HIV. In “recognition of vulnerability,” participants described reasons for being worried about getting HIV but said no or limited action was taken to reduce their perceived vulnerability. Participants with “no rationalization or action” did not describe any HIV worry or did not engage in HIV risk reduction behaviours.

**Conclusions:**

Women who are at substantial risk of acquiring HIV may underestimate their actual risk. Yet, others who accurately understand their HIV risk may be unable to act on their concerns. Perceived HIV risk and risk rationalization are important concepts to explore in risk reduction counselling to increase the use of HIV prevention strategies among women at risk of HIV.

## Introduction

An individual's perception of risk for a particular illness is a core construct in many behaviour change theories in public health [1, 2]. Although perception of risk is one of several theoretical constructs that may influence behaviour change, the main premise is that individuals who believe they are at risk of acquiring an illness may be more likely to engage in behaviours to reduce that risk. Conversely, individuals who do not believe they are at risk may be unlikely to modify their risk behaviours. Perception of HIV risk has been studied extensively in the field of HIV prevention [3–13]. Yet, limited data are available on individuals’ perceptions of their HIV risk around the time of HIV infection.

In FEM-PrEP [14], we explored perceptions of HIV risk among participants at multiple times throughout the trial. We also retrospectively explored HIV worry among participants who seroconverted. We wanted to learn more about how women who were at substantial HIV risk understood and contextualized their HIV risk, as these beliefs may influence use of HIV risk reduction methods. In this article, we focus on HIV risk perceptions and worry about HIV acquisition among participants who seroconverted during the trial from two sites – Bondo, Kenya, and Pretoria, South Africa. We first report how participants who seroconverted perceived their HIV risk shortly before they became infected with HIV. We then describe the context surrounding perceived HIV risk among participants by describing reasons for worrying or not worrying about acquiring HIV prior to becoming infected.

## Methods

### Overview of the FEM-PrEP clinical trial

FEM-PrEP was a phase III, placebo-controlled clinical trial to assess the safety and effectiveness of once-daily, oral emtricitabine (FTC) and tenofovir disoproxil fumarate (TDF) for the prevention of HIV among women. The trial was conducted in Bondo, Kenya; in Bloemfontein and Pretoria, South Africa; and in Arusha, Tanzania. Details of the trial are described elsewhere [14]. Briefly, women considered to be at higher risk for HIV acquisition (i.e. those who either had vaginal sex at least once within the past two weeks or had sex with more than one sexual partner in the past month) were randomized to once-daily FTC-TDF or placebo. Participants were asked to take their assigned study product for 52 weeks and to attend clinic visits every four weeks for 60 weeks. Among other trial procedures, participants were tested for HIV and received risk reduction counselling, a free supply of condoms and treatment for any curable sexually transmitted infections at each visit. Sixty-eight participants (34 from Pretoria, 27 from Bondo and 7 from Bloemfontein) became infected with HIV on or before week 52. Participants who seroconverted were asked to attend clinic visits one week after their HIV diagnosis visit and every four weeks for 52 weeks.

Qualitative research on adherence, sexual behaviours and HIV risk perceptions was embedded within the clinical trial protocol at the Bondo, Pretoria and Arusha sites. The trial began in July 2009 and closed early in April 2011 because of lack of effectiveness [15]. At closure, the Bondo and Pretoria sites were fully enrolled, and most participants had completed or nearly completed their follow-up visits; the Arusha site had recently initiated; and the Bloemfontein site was still enrolling, and no participant there had completed all follow-up visits.

### Data collection

#### Quantitative interviews

We conducted a quantitative, face-to-face interview on perception of HIV risk with all participants at enrolment and at quarterly follow-up visits. We asked participants to report on the likelihood that they would become infected with HIV in the next four weeks. Response options were 1) no chance – no possibility of becoming infected with HIV; 2) a small chance – could happen but not likely; 3) a moderate chance – some possibility of becoming infected; and 4) a high chance – likely to become infected. The interview was conducted at the beginning of the study visit, prior to participants learning their HIV test results; the questions on risk perceptions were no longer asked once a participant had seroconverted. We also asked demographic, sexual behaviour and relationship questions at baseline through a quantitative face-to-face interview. All quantitative interviews were conducted at the study clinic and in the local language (Setswana in Pretoria, and Dholuo or Kiswahili in Bondo) or in English by trained, local, female staff interviewers.

#### Qualitative interviews

We conducted semi-structured interviews (SSIs) with participants who seroconverted at the Bondo and Pretoria sites to retrospectively explore, among other topics, HIV worry – a measure of perceived vulnerability [16] and a concept similar to HIV risk perception [17, 18]. All participants who seroconverted at these sites were invited to participate in SSIs at weeks 1, 4, and 8 after their HIV diagnosis visit. We chose these weeks based on previous experience in conducting interviews at similar time points with individuals diagnosed with acute HIV infection in Malawi [19]. In the SSIs at week 1, participants were asked to describe whether they worried (prior to their diagnosis) about becoming infected with HIV, the reasons why they did or did not worry and whether they took any actions because of their perceived vulnerability. Participants who missed their week 1 interview or did not discuss HIV worry during their week 1 interview were asked about HIV worry at the subsequent week 4 or week 8 interview. The SSIs were audio-recorded if the participant agreed; detailed notes were taken otherwise. Trained, local female interviewers, who were different from those who conducted the quantitative interviews, conducted the SSIs at the study clinic in the local language or in English.

### Data analysis

Among the 61 participants who seroconverted in Bondo and Pretoria, we calculated the frequencies of their responses on perceived HIV risk that were reported at the study visit that was conducted closest to, but before, HIV acquisition (based on the estimated window dates of HIV infection [14, 20]). Of these participants, 56 (32 from Pretoria and 24 from Bondo) participated in at least one SSI, for an inclusion rate of 94% for Pretoria and 89% for Bondo. No participant refused to participate in an SSI; missed interviews occurred when participants did not return for their follow-up study visits.

Applied thematic analysis was used to analyse the qualitative data [21]. Interviewers simultaneously transcribed and translated audio-recorded SSIs into word-for-word English transcripts (while maintaining the overall meaning of the participants’ narratives) following a transcription protocol [22]; 51 participants agreed for at least one of their interviews to be audio-recorded. For SSIs that were not audio-recorded, interviewers expanded their detailed notes immediately after the interview and categorized the discussion by content area.

To better understand the overall context of HIV worry or non-worry, we reviewed all direct responses to questions on HIV worry as well as other related behavioural data provided by each participant in the SSIs. Using NVivo 9 [23], two qualitative analysts applied a content code on “HIV worry/non-worry” to capture text from the SSIs in which participants spoke about whether or not they worried about HIV prior to their HIV diagnosis and to capture other related narratives. Inter-coder reliability (ICR) was assessed throughout the content-coding process; 22 transcripts were assessed across the week 1, 4 and 8 interviews. Independent coding occurred after the first three ICR sessions, at which point the analysts had consistently and repeatedly reached agreement on almost all code applications. The remaining four ICR sessions were interspersed throughout the coding period. At each ICR session, coding discrepancies were discussed and resolved, and the codebook and previously coded transcripts were revised if necessary.

When coding was completed, the content-coding reports were reviewed, and a matrix-based approach was used to display reasons that participants gave for worrying or not worrying about acquiring HIV. Using the matrix, we identified themes such as “having only one partner” and “trusting partner” that led participants to worry or not worry about acquiring HIV. The frequencies of each theme and representative illustrative quotes were then summarized in a data memo. Based on the similarities of the themes and perceived vulnerability, we grouped each theme into broad risk rationalization categories. Data from five participants (three from Pretoria and two from Bondo) were excluded because we were unable to discern any meaning about vulnerability from their narratives; thus, the total SSI sample size is 51 (29 from Pretoria and 22 from Bondo).

### Ethics

All associated ethics and regulatory committees approved the trial. All participants provided their informed consent to participate in the SSIs when they signed the clinical trial enrolment consent form; before each SSI, participants were verbally asked if they were still willing to be interviewed.

## Results

### Baseline characteristics among participants who seroconverted in Bondo and Pretoria

Twenty-eight (82%) of the participants in Pretoria were under 25 years of age, compared with 12 (44%) of the participants in Bondo (Table 1). Participants in Pretoria also had more years of schooling (mean: 10.7 years) than participants in Bondo (mean: 8.2 years). Occupations also varied among the sites. A high percentage of participants in Bondo were daily wage earners (63%, *n =* 17), such as through employment at a restaurant or bar or by selling fish; most participants in Pretoria (65%, *n =* 22) reported not having an occupation. Table 1 also describes sexual behaviours and beliefs about sexual partners reported during the quantitative questionnaire that was conducted at enrolment. In the SSIs, 41 (73%) of the participants (*n =* 16 in Bondo; *n =* 25 in Pretoria) described having only one sexual partner around the time of HIV infection.

**Table 1:** Baseline characteristics among participants who seroconverted in Bondo, Kenya, and Pretoria, South Africa

Variables^a^	Pretoria (*n=*34)	Bondo (*n=*27)
Age in years		
<25	28 (82)	12 (44)
≥25	6 (18)	15 (56)
Education		
Finished primary school	28 (82)	4 (15)
Education in years^b^	10.7 (11), 4–14	8.2 (8), 3–16
Married	1 (3)	16 (59)
Occupation		
None	22 (65)	5 (19)
Student	10 (29)	2 (7)
Daily wage job	2 (6)	17 (63)
Others^c^	0	3 (11)
**Sexual behaviours at enrolment** ^**d**^		
Type of partner		
Primary partner *only*	27 (79)	16 (59)
More than one sexual partner^e^	7 (21)	11 (41)
No. of vaginal sex partners in past 7 days^b^	1.0 (1), 0–2	1.0 (1), 0–2
No. of vaginal sex acts with primary partner in past 7 days^b,f^	3.4 (2), 0–12	2.9 (2), 0–8
No. of vaginal sex acts with other partner in past 7 days^b,g^	0.3 (0), 0–4	0.5 (0), 0–6
Had sex without condom with primary partner^f^	15/34 (44)	22/27 (82)
Had sex without condom with other partner^g^	0/6 (0)	3/11 (27)
Had anal sex with primary or other partner	0/33 (0)	1/27 (4)
Exchanged sex for money or goods^g^	3/6 (50)	5/11 (46)
**Beliefs about sexual partner at enrolment**		
Believed primary partner has HIV^f^		
No	22 (65)	14 (52)
Yes	1 (3)	0
Do not know	11 (32)	13 (48)
Believed primary partner had vaginal or anal sex with other sexual partners in the past 4 weeks^f^		
No	13 (38)	3 (11)
Might/yes	2 (6)	10 (37)
Do not know	19 (56)	14 (52)
Believed other sexual partner in the past 4 weeks had HIV^g^	(*n=*6)	(*n=*11)
No	2 (33)	0
Yes	0	0
Do not know	4 (67)	11 (100)

a *n* (%) reported unless specified

b mean (median), range

c all participants in other category in Bondo are farmers

d reported for the past 4 weeks unless otherwise noted

e primary partner plus at least one other partner or two or more other partners

f among participants who reported having a primary partner

g among participants who reported having other partners.

### HIV risk perceptions and worry

**Figure 1:**
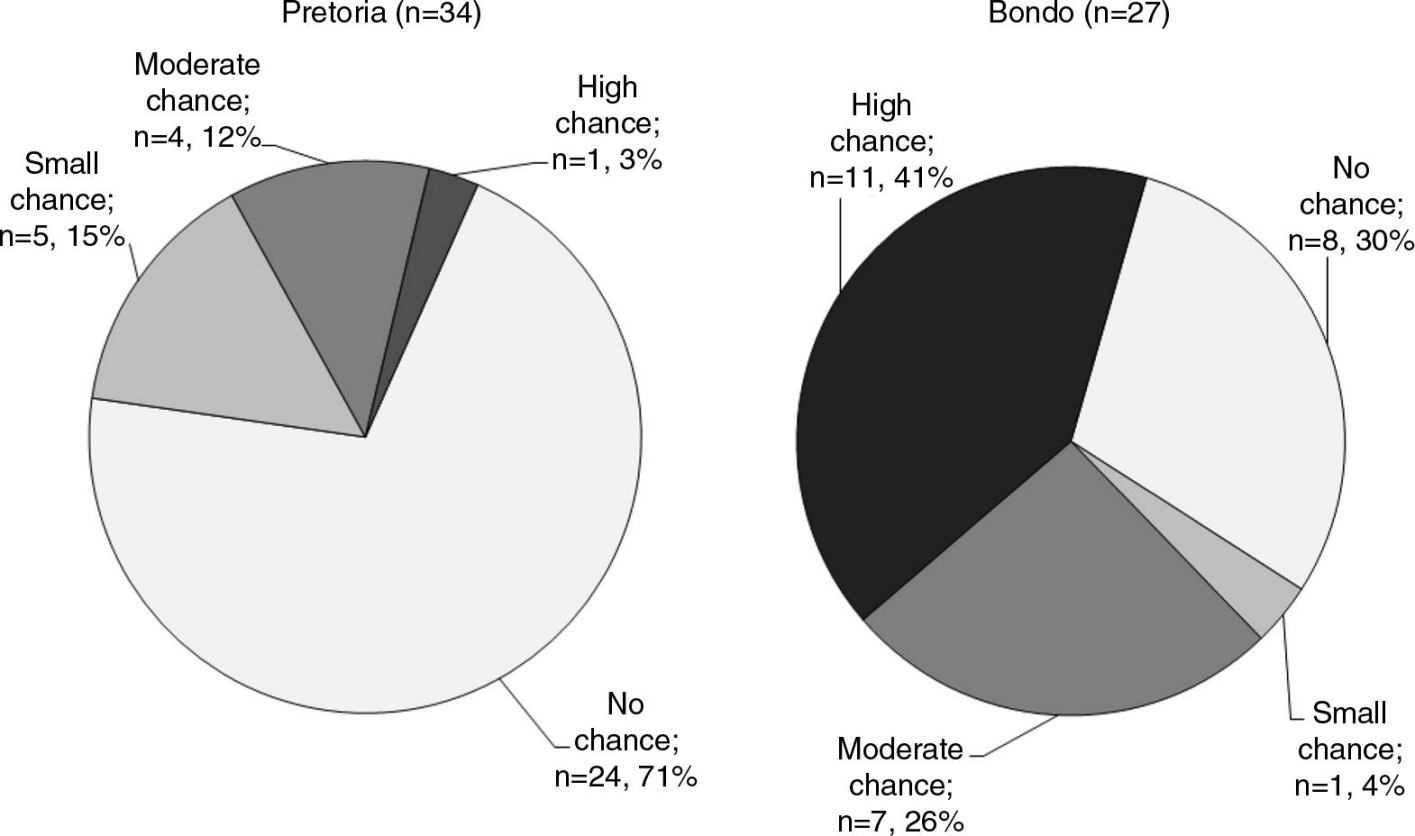
Perception of HIV risk reported at the visit conducted closest to, but before, HIV acquisition, by site.

Fifty-two percent (*n =* 32) of participants reported in the quantitative interview, which was conducted at the visit closest to (but before) becoming HIV infected, that they had no chance of acquiring HIV in the next four weeks. Perception of HIV risk varied significantly between the two sites (*p <* 0.0001, using a chi-square test.) (Figure 1). Prior to becoming infected with HIV, the participants in Pretoria who seroconverted had generally perceived themselves to be at less risk than had the participants in Bondo who seroconverted; 24 (71%) of the participants in Pretoria compared with 8 (30%) of the participants in Bondo indicated that they had no chance of acquiring HIV in the next four weeks. Conversely, 11 (41%) of the participants in Bondo compared with one participant (3%) in Pretoria believed they had a high chance of acquiring HIV.

The findings about HIV worry from the SSIs were similar to the findings about risk perceptions from the quantitative interviews. Most participants in Pretoria (*n =* 25) said that they did not worry about getting HIV prior to their HIV diagnosis, but many participants in Bondo (*n =* 10) said they did. Additionally, 24 of the participants (mostly from Pretoria) in the SSIs said they *never* thought they would get infected with HIV.

### Risk rationalizations

We identified and labelled four processes of risk rationalization from the thematic analysis of the 51 participant narratives in the SSIs: 1) protective behaviour, 2) protective reasoning, 3) recognition of vulnerability and 4) no rationalization or action. These processes of risk rationalization illuminate participants’ perceptions of their risk context and, for some, how risk management strategies, or a lack thereof, influenced their sense of vulnerability towards acquiring HIV.

#### Protective behaviour

In the SSIs, 14 participants (nine from Pretoria and five from Bondo) described that they engaged in well-known HIV risk reduction practices; these actions made them feel not vulnerable to HIV, and therefore they did not worry about acquiring the virus. SSI narratives that focused on protective behaviours were mostly by participants in Pretoria who reported in the quantitative interviews that they had no chance of acquiring HIV.

Condom use was one of two commonly mentioned protective behaviours in the SSIs that led many of these participants (*n=*7) to not worry about getting HIV. For example, a participant from Pretoria was confident in her reasoning for previously not worrying about getting HIV: “We used to use a condom when we had sex. Ja. So I was sure that whatever he may do, I know I protect myself.” Yet, later in the interview she described inconsistent condom use, which she believed led her to become infected: “We woke up at night and he wanted us to have sex. I never thought about a condom … [but] when we were using a condom, we were okay.”

Having only one sexual partner was the other protective behaviour frequently mentioned (*n=*9). A participant from Pretoria said: “I never thought I would get infected with HIV. My own behaviour did not give it a chance. I only had one partner, and I never slept around.” Another participant from Pretoria said: “I never got worried … I knew myself.” She elaborated on her reason for not previously worrying about HIV when describing her response to learning she had HIV: “I never thought I could be infected by this … [because] of the way I behaved myself … My condition was good. I behaved myself very well. I have one partner.”

Only one participant, who was from Bondo, mentioned that taking the study pill led her to not worry about acquiring HIV. In her SSIs, she described that the purpose of the two-arm study was to assess whether FTC-TDF could reduce the risk of HIV acquisition, but she hoped she was assigned FTC-TDF because she believed it would work for HIV prevention. Prior to seroconversion, she believed she was taking FTC-TDF because she had repeated HIV-negative test results. After seroconversion, however, she believed she was assigned the placebo because she acquired HIV.

#### Protective reasoning

Narratives in the SSIs suggested that 23 participants (16 from Pretoria and 7 from Bondo) actively reflected upon their HIV risk but rationalized, because of certain events or beliefs, that they were not vulnerable to acquiring HIV, and therefore they did not worry about the possibility of infection. In most cases, this rationalization led participants to think that continued risk reduction behaviours, such as using condoms, were unnecessary. The majority of these SSI narratives were from participants in Pretoria who reported no chance of acquiring HIV in their quantitative interviews.

Perceptions about HIV testing influenced SSI participants’ protective reasoning. Many participants (*n=*10) described that having multiple HIV-negative test results before or during the trial led them to not worry about acquiring HIV. A participant from Bondo commented: “I have never thought of that [worry about HIV]. Depending on the days that I have always come for my visits, I always tested negative. Even when I came I never thought I would test HIV positive.” Some participants (*n=*4) described that knowing their partner's previous HIV-negative status made them not worry. A participant from Pretoria explained:

I don't know what to say, but I did not expect that at this moment I would be HIV-positive … The person I was involved with got tested and the results came out negative. I was also getting tested and I was negative so I did not expect it. I don't know – maybe he hid it from me because you can be tested and there is still a window period.

Assuming to know a partner's HIV status based on one's own HIV status was also mentioned by some participants (*n=*3) in Pretoria: “I never thought that my boyfriend could be HIV infected. I knew that I was not infected with HIV, so I never thought that he might be sick.”

Trust was another concept that informed several participants’ protective reasoning (*n=*9). Trust related to HIV worry was multifaceted, was often interwoven with HIV testing and was described primarily by participants in Pretoria. Some participants (*n=*3) described trusting their partners because they assumed they were in monogamous relationships. A participant from Pretoria who had multiple non-concurrent relationships said: “I trusted my boyfriends … because I did not see them cheating on me.” Furthermore, two participants also believed their partners would not infect them – a participant from Bondo explained: “I also have one sexual partner, whom I trusted and knew cannot make me get there [be HIV positive]. And, I also would not like to put him there.”

One participant from Pretoria explained that trust was built over time through repeated, back-to-back HIV-negative test results: “Because most of the time I knew myself … and then I went [for HIV testing] after three months – isn't it that they say window period? They told me to come after three months. I went back; then that's when we trusted each other.” Later in the interview, the participant further described how this trust halted condom use with her partner:

It's that I am with one person. The first time we had sex we were using condoms. And then he told me that we have to stop using condoms; but before [we stop] we have to test. We tested many times and they were negative. So, that's when I started trusting him, and then I ended up being positive.

For some participants (*n=*3), trust was also formed based on assumptions they had about their partners’ sexual behaviours given their own sexual behaviours. For example, a participant from Pretoria described that she did not worry about getting HIV because she was faithful and believed her partner was as well: “I knew I was behaving well and I thought he was behaving well too.”

Narratives from three participants in Bondo included statements that demonstrated both protective behaviours and protective reasoning. These participants were grouped in both categories above. All other participants were counted in only one of the risk rationalization processes.

#### Recognition of vulnerability

Eleven participants (1 from Pretoria and 10 from Bondo) described in the SSIs specific reasons why they worried about getting HIV and suggested that they were unable to consistently engage in risk reduction behaviours to reduce their perceived vulnerability. Most of these participants perceived their risk to be moderate or high in the quantitative interviews.

Participants described two main reasons for their HIV worry: 1) knowing that their partners have other sexual partners, or being uncertain of their partners’ monogamy (*n=*7); and 2) not knowing their partners’ HIV status (*n=*6). A participant from Bondo explained: “That [HIV] is something I knew was there and I could get it at any time … because I know my status but I don't know his status. I don't know his sexual behaviour. I just know my sexual behaviour.”

All participants described infrequent or inconsistent condom use (*n=*7) – or never using condoms at all (*n=*4). A participant from Bondo explained that not using a condom within the overall context of uncertainty about her partner's fidelity led her to worry: “I had thought of that [worry about HIV] because my husband and I had got tested a long time ago and we do not use condoms, but I don't know how his movements are and if he has other sexual partners.” Many participants (*n=*6) described that their sexual partners disliked using condoms and they were therefore using them infrequently, if at all. Cultural traditions and community gender roles, including limited sexual decision-making power among women, were also described by a participant from Bondo as a reason why she worried about getting HIV:

It was worrying me based on how I was told that woman [her husband's other wife] was behaving … I had even told him, I told him clearly that ‘imagine you will get infected with a disease that you don't even know’. I used to sit down to talk softly with my husband, but he could not listen to my opinion because you know I am a woman and he is a man. So our thoughts could not rhyme. So I was really worried.

Earlier in the interview, the participant explained that because of Luo customs, she was unable to use a condom with her husband during certain rituals.

#### No rationalization or action

Narratives from six participants (three from Pretoria and three from Bondo) lacked any mention of prior rationalization of HIV risk or examples of actions to reduce the chance of HIV acquisition. Four participants described that they never thought they would become infected; two of the four described being in long term relationships.

## Discussion

During the FEM-PrEP clinical trial, we were able to assess participants’ perceptions of their HIV risk during the period shortly before they became infected with HIV. We were also able to gather rich qualitative data on participants’ rationales for having previously worried or not worried about acquiring HIV. In the quantitative interviews, more than half of the participants who seroconverted (52%) did not perceive that they had been at any risk for HIV around the time they became infected. The majority of these participants were from Pretoria. Similarly, most participants in the SSIs who described not worrying about acquiring HIV were from Pretoria. Generally, SSI narratives suggested that participants’ own sexual behaviours and beliefs influenced their protective behaviours and protective reasoning and led these participants to not worry about acquiring HIV. Together, these findings suggest that many women who are at substantial risk of acquiring HIV may not perceive their current sexual context to be risky and therefore may underestimate their actual risk.

Several factors within the participants’ broader environment may have influenced the protective behaviours and protective reasoning they described in the SSIs, potentially leading them to believe that their current sexual context was not risky. First, the dropping rates of HIV infection in some of the study countries around the time of trial initiation may have left participants feeling less at risk or vulnerable. For instance, HIV incidence in South Africa had declined by more than 25% between 2001 and 2009. Also, prevalence in Kenya had declined to 5% by 2006 from 14% in the mid-1990s [24]; however, in 2008–2009, the prevalence was still high at 13.9% in Nyanza Province, where Bondo is located [25]. Second, we provided risk reduction counselling and HIV testing at each study visit as part of trial procedures. Receiving frequent HIV-negative test results influenced many participants’ rationalizations that they were not vulnerable to HIV and potentially led them to believe or reaffirmed their beliefs that they were not engaging in risky sexual behaviours. Third, based on the theory of cognitive dissonance [26], participants may have believed they were at risk of HIV but rationalized that they were not at elevated risk to reduce or avoid the discomfort associated with feeling at risk. Similar findings, albeit with different rationalizations, were found in a study among female sex workers in Nigeria [27]. Fourth, participants may have believed they truly were not at risk because they were following two of the three prevention steps in the widely promoted ABCs of HIV prevention – B for “Be Faithful” and C for “Use Condoms” (albeit they used condoms inconsistently) – and either assumed their partners were also following such guidelines or did not consider their partners’ behaviour. Based on these data, we conducted follow-up interviews with participants in Bondo and Pretoria to explore the broader context that may influence perceptions of faithfulness and trust; these data are to be presented elsewhere.

In contrast to the participants who may have underestimated their HIV risk, many participants who seroconverted during the FEM-PrEP trial (48%) appeared to accurately understand their HIV risk but were unable to act on their concerns. Most of these participants were from Bondo, and their beliefs about their partners’ risky sexual behaviour, as described in the SSIs, strongly influenced their own higher perception of vulnerability. Concerns about spousal infidelity have also been shown to influence HIV worry among women, and men, in Malawi [28]. Gender-based power dynamics, as have frequently been documented in the literature, often impede a woman's ability to engage in risk reduction behaviours, particularly using condoms with her primary partner, in marriage, or within inherited relationships (which are common among widows in Bondo) [29–38].

Because we collected quantitative data on risk perceptions during participants’ quarterly visits, responses may have reflected participants’ perceptions for up to 12 weeks before they became infected with HIV. It is possible that perceptions of risk for some participants may have increased or decreased immediately prior to HIV infection given a change in their risk context. However, in the SSIs, most participants said that their sexual behaviour and that of their partners remained similar in the months prior to HIV infection. Furthermore, because of the likely emotional response to learning one's HIV status, recall bias or a bias related to self-protection may also have affected how some participants retrospectively reflected on their HIV worry prior to diagnosis.

Based on our findings, we recommend that future risk reduction interventions consider how to incorporate women's HIV risk perceptions, particularly their risk rationalizations, into the HIV prevention discourse [39, 40], as they are likely important precursors for adopting any risk reduction measures. This is especially true for women who are similar to the study participants in the Pretoria site, where many participants generally had low perceptions of HIV risk and low perceived vulnerability towards acquiring HIV, but where a high overall HIV incidence (6.0 per 100 person years in the placebo arm) was reported in the trial [14]. A significant challenge in doing so, however, will be aligning women's feelings about their risk with their actual level of risk [41]. For women who are not in a recognized serodiscordant relationship, exposure to HIV is often unknown. Moreover, many women may have little HIV risk or not be at risk of HIV at all (e.g. when partners are HIV negative, and in monogamous relationships between HIV-negative individuals). Therefore, in HIV risk reduction counselling and other HIV prevention programmes, an intricate balance is needed to simultaneously 1) encourage women to more appropriately evaluate the potential likelihood of becoming infected with HIV; 2) ensure that couple harmony is maintained [42] (e.g. not introduce unnecessary conflict within the relationship when HIV risk is actually low) and the relationship is preserved (e.g. among women who want to or who must remain with their partners because of social or financial consequences); and 3) prevent other adverse consequences, such as denial and defensive coping [27], of asking women who may be at actual HIV risk to reassess their potential risk.

For women in risk situations similar to those of many of the participants in Bondo, where perceived HIV risk, vulnerability towards HIV and HIV incidence (4.7 per 100 person years in the placebo arm [14]) were generally high, a woman-controlled HIV risk reduction method, such as pre-exposure prophylaxis (PrEP) and potentially microbicides and the vaginal ring (pending current trial outcomes [43–45]), may be more realistic. Moreover, interventions that focus on 1) empowering women and increasing economic independence while transforming gender norms, 2) encouraging safer sexual health practices within marriage and 3) incorporating male involvement into the promotion and use of women-centred approaches [34, 36, 37, 46–48] must continue and intensify.

## Conclusions

In moving forward with HIV prevention among women, whether in PrEP demonstration projects or in non-biomedical HIV prevention programmes, perceived HIV risk and risk rationalization are important concepts to explore. Understanding and responding to women's perceptions and rationalizations about risk could enhance the use of risk reduction methods, particularly in populations similar to those in the FEM-PrEP clinical trial.
